# Influence of Cobalamin on Arsenic Metabolism in Bangladesh

**DOI:** 10.1289/ehp.0900734

**Published:** 2009-07-31

**Authors:** Megan N. Hall, Xinhua Liu, Vesna Slavkovich, Vesna Ilievski, Zhongyuan Mi, Shafiul Alam, Pam Factor-Litvak, Habibul Ahsan, Joseph H. Graziano, Mary V. Gamble

**Affiliations:** 1 Department of Epidemiology; 2 Department of Biostatistics and; 3 Department of Environmental Health Sciences, Mailman School of Public Health, Columbia University, New York, New York, USA; 4 Columbia University Arsenic Project in Bangladesh, Dhaka, Bangladesh; 5 Department of Health Studies and; 6 Department of Medicine and Human Genetics and Cancer Research Center, University of Chicago, Chicago, Illinois, USA

**Keywords:** arsenic, Bangladesh, cobalamin, creatinine, dimethylarsinic acid, folate, homocysteine, monomethylarsonic acid, one-carbon metabolism

## Abstract

**Background:**

Arsenic is a carcinogen to which 35 million people in Bangladesh are chronically exposed. The enzymatic transfer of methyl groups to inorganic As (iAs) generates monomethylarsonic (MMA) and dimethylarsinic acids (DMA) and facilitates urinary As (uAs) elimination. This process is dependent on one-carbon metabolism, a pathway in which folate and cobalamin have essential roles in the recruitment and transfer of methyl groups. Although DMA^V^ is the least toxic metabolite, increasing evidence suggests that MMA^III^ may be the most cytotoxic and genotoxic As intermediary metabolite.

**Objective:**

We examined the associations between plasma cobalamin and uAs metabolites.

**Methods:**

We conducted a cross-sectional study of 778 Bangladeshi adults in which we over-sampled cobalamin-deficient participants. Participants provided blood samples for the measurement of plasma cobalamin and urine specimens for As measurements.

**Results:**

Cobalamin was inversely associated with the proportion of total uAs excreted as iAs (%iAs) [unstandardized regression coefficient (b) = –0.10; 95% confidence interval (CI), −0.17 to −0.02; *p* = 0.01] and positively associated with %MMA (b = 0.12; 95% CI, 0.05 to 0.20; *p* = 0.001). Both of these associations were stronger among folate-sufficient participants (%iAs: b = −0.17; 95% CI, −0.30 to −0.03; *p* = 0.02. %MMA: b = 0.20; 95% CI, 0.11 to 0.30; *p* < 0.0001), and the differences by folate status were statistically significant.

**Conclusions:**

In this group of Bangladeshi adults, cobalamin appeared to facilitate the first As methylation step among folate-sufficient individuals. Given the toxicity of MMA^III^, our findings suggest that in contrast to folate, cobalamin may not favorably influence As metabolism.

Ingestion of arsenic through contaminated drinking water is a major health concern in Bangladesh, where approximately 35 million people are chronically exposed (Kinniburgh and Smedley 2001). This exposure has been linked to increased risks of cancers of the skin, bladder, liver, and lung [California Environmental Protection Agency (EPA) 2004; Navarro et al. 2007], as well as ischemic heart disease and neurologic impairments (Navarro et al. 2007).

Metabolism of As occurs through a series of reduction and methylation reactions (Figure 1). The methylation steps are catalyzed by arsenic methyltransferase (AS3MT), with *S*-adenosylmethionine (SAM) serving as the methyl donor (Lin et al. 2002; Marafante and Vahter 1984). Inorganic arsenite is first methylated to monomethylarsonic acid (MMA^V^). Upon reduction to MMA^III^, it then undergoes a second methylation to dimethylarsinic acid (DMA^V^). Methylation has generally been considered a detoxification pathway that generates the least toxic organic mammalian arsenical metabolite, DMA^V^, and facilitates urinary excretion (Vahter and Marafante 1987). However, increasing evidence suggests that MMA^III^ is a highly cytotoxic and genotoxic intermediate (Styblo et al. 2002; Wang et al. 2007).

Folate, cobalamin (vitamin B12), and vitamin B6 are required for the recruitment and transfer of methyl groups in one-carbon metabolism. Other nutrients, including protein, betaine, and choline, also contribute to the availability of methyl groups ultimately used in SAM biosynthesis. Arsenic retention in tissues is increased with dietary methyl donor deficiency (Vahter and Marafante 1987), and urinary As (uAs) excretion is decreased in folate deficiency (Spiegelstein et al. 2003). Our previous work has shown that individual variation in the ability to methylate As is associated with folate status (Gamble et al. 2005b; 2006; 2007). However, few investigations of the association between cobalamin and As metabolism have been conducted, despite its critical role as an essential cofactor for methionine synthase, which catalyzes the remethylation of homocysteine to methionine, a critical step in SAM biosynthesis. Zakharyan and Aposhian (1999) reported that *in vitro* incubation of methylcobalamin (CH_3_B_12_) with arsenite in the presence of a reducing agent can produce MMA and trace amounts of DMA nonenzymatically. We observed no association between plasma cobalamin and uAs metabolites in our previous cross-sectional study of 300 adults (Gamble et al. 2005b). Because the primary aim of that study was to examine the influence of folate on As methylation, we excluded cobalamin-deficient participants, thereby restricting the range of plasma cobalamin values.

To examine the influence of a full range of plasma cobalamin concentrations on As metabolism, we conducted a cross-sectional study in which we oversampled cobalamin-deficient participants (plasma cobalamin < 185 pmol/L). We also set out to replicate the *in vitro* work of Zakharyan and Aposhian (1999) in a slightly modified system, as this finding would contribute to our understanding of the role that cobalamin might play in influencing the metabolism of As.

## Materials and Methods

### Study participants, procedure, and ethics

Using biologic samples from our original cross-sectional study of a random sample of 1,650 adults [the Nutritional Influences on Arsenic Toxicity study (Gamble et al. 2005a)], we analyzed uAs metabolites for all of the cobalamin-deficient participants (*n* = 412 with plasma cobalamin < 185 pmol/L). For efficiency, we combined these individuals with an additional 366 nondeficient participants who already had uAs metabolite data, to incorporate a broad range of cobalamin nutritional status. This process yielded a total of 778 participants. All participants had provided blood and urine samples, and demographic information was obtained from a baseline survey. All study subjects gave informed oral consent to Bangladeshi field staff physicians before participating in the study. The research protocol was approved by the Institutional Review Board of Columbia University and the Bangladesh Medical Research Council.

### Collection of biospecimens

Blood samples were collected by venipuncture from participants who had been sitting for 10–15 min. The samples were collected into EDTA-containing tubes that were immediately placed in IsoRack cool packs (Brinkmann Instruments, Westbury, NY, USA). Samples were transported by hand (in coolers containing additional ice packs) to our field clinic in Araihazar within 4 hr of collection. The samples were then centrifuged at 4°C to separate plasma and cells. Plasma was stored at −80°C and shipped frozen on dry ice to Columbia University for analysis. Urine samples were collected in 50-mL acid-washed tubes, frozen at −20°C, and shipped on dry ice.

### Measurement of plasma nutrients

Plasma cobalamin and folate were analyzed by radioimmunoassay (Quantaphase II; Bio-Rad Laboratories, Richmond, CA, USA) as previously described (Gamble et al. 2005a; Pfeiffer et al. 2005). The within-day coefficient of variation (CV) was 3% for folate and 4% for cobalamin. The between-day CV was 11% for folate and 8% for cobalamin. We measured plasma total homocysteine (tHcys) using high-performance liquid chromatography (HPLC) with fluorescence detection (Pfeiffer et al. 1999) as described previously (Gamble et al. 2005a).

### Well water As

We used graphite furnace atomic absorption (GFAA), with a detection limit of 5 μg/L, to measure total As in well-water samples. Samples found to have a concentration < 5 μg/L were reanalyzed by inductively coupled plasma-mass spectrometry (ICP-MS), for which the detection limit was 0.1 μg/L (Cheng et al. 2004).

### Total uAs and creatinine

Total uAs was measured in the Columbia University Trace Metals Core Laboratory by GFAA spectrometry using the Analyst 600 graphite furnace system (PerkinElmer, Shelton, CT, USA), according to the method of Nixon et al. (1991). Our laboratory participates in a quality control program organized by Philippe Weber at the Quebec Toxicology Center in Quebec, Canada. Intraclass correlation coefficients between our laboratory’s values and samples calibrated at the Quebec laboratory were 0.99. We used a method based on the Jaffe reaction (Slot 1965) to measure urinary creatinine concentrations.

### uAs metabolites

As metabolites were speciated using HPLC separation of arsenobetaine (AsB), arsenocholine (AsC), arsenate, arsenite, MMA, and DMA, followed by detection using ICP-MS. We calculated the percentages of iAs (iAs^III+V^), MMA (MMA^III+V^), and DMA (DMA^III+V^) after subtracting AsC and AsB from the total.

### Assay for *in vitro* generation of MMA and DMA by methylcobalamin and iAs^III^

Because our epidemiologic data suggested a role for cobalamin in the generation of MMA, but not DMA, we sought to replicate the findings of Zakharyan and Aposhian (1999) that suggested methylcobalamin could non-enzymatically transfer its methyl group to arsenite. We modified their system so as to not include SAM. Arsenite^III^, cobalamin, CH_3_B_12_, L-glutathione (GSH), MMA, and DMA were purchased from Sigma (St. Louis, MO, USA). Arsenate was purchased from PerkinElmer. All other chemicals were analytical reagent grade. Water was distilled and deionized. The assay for nonenzymatic arsenite methylation was done using 0.05 M Tris-HCl buffer at various pH values containing 10 mM GSH. At each pH, we used molar ratios of CH_3_B_12_:arsenite of 1:5, 3:5, and 5:5. We performed three replicates for every combination of pH and molar ratio. The reaction mixture was incubated for 1.5, 3, 6, 9, 12, and 30 hr at 37°C. At each time point we aliquoted 50 μL of the mixture, diluted it with mobile phase (10 mM ammonium phosphate plus 10 mM ammonium nitrate), injected it onto the HPLC column, and detected concentrations of MMA, DMA, As^III^, and As^V^ by ICP-MS with dynamic reaction cell technology (ICP-MS-DRC). Calibration standards of a mixture of As metabolites were similarly processed. ICP-MS-DRC coupled to HPLC separates and detects 7 As metabolites chromatographically separated by anion exchange with the use of a PRP-X100 column (Hamilton Co, Reno, NV, USA).

### Statistical analysis

Plasma nutrients and uAs metabolites were not normally distributed. We therefore used nonparametric tests where appropriate and natural log transformations that may either create approximately normal distributions for dependent variables in linear regression analysis, improve linearity in the relationship between an independent and dependent variable, or reduce the impact of extreme values of an independent variable in fitting linear models. We calculated descriptive statistics separately by sex for general characteristics as well as for plasma nutrient and uAs metabolite concentrations. We tested for sex differences using the Wilcoxon rank-sum test for continuous variables and the chi-square test for categorical variables. The correlations between plasma cobalamin and covariates and between cobalamin and the percentage of As metabolites in urine were examined using Spearman’s correlation coefficients. We used linear regression models to examine associations between plasma cobalamin and the outcome variables %iAs, %MMA, and %DMA in urine. Potential confounders considered for inclusion in the regression models were age, sex, urinary creatinine, body mass index (BMI), and total uAs. We used a Wald test to detect differences in the covariate-adjusted associations between cobalamin and uAs metabolites by sex, folate status, or water As status. We ran separate linear regression models to examine the covariate-adjusted associations between plasma cobalamin and each of the uAs metabolites and to examine the differences in these associations by sex, folate status, or water As status. Analyses were performed using SAS 9.1 (SAS Institute Inc., Cary, NC, USA). All statistical tests were two-sided with a significance level of 0.05. The As metabolite concentrations detected in the nonenzymatic laboratory assay experiments were expressed as a percentage of total detected metabolites.

## Results

### Cross-sectional study of cobalamin and uAs metabolites

The general characteristics and plasma nutrient concentrations of the study participants by sex are shown in Table 1. The men in this sample were significantly older than the women and had a lower mean BMI. Nearly 60% of men were current cigarette smokers compared with 3% of women. Both women (30.6%) and men (40.9%) had a high prevalence of current betel nut use. Water As concentrations of the wells that served as the primary source of drinking water were above the Bangladeshi standard (50 μg/L) for 60% of women and 58% of men. The water As concentrations were above the World Health Organization standard (10 μg/L) for 81% of women and 83% of men.

Because we oversampled cobalamin- deficient participants, a large proportion of the sample had cobalamin deficiency: 57.6% of women and 46.8% of men had < 185 pmol/L. We used cutoff values of < 9.0 nmol/L for folate deficiency (Christenson et al. 1985), and ≥ 10.4 for women and ≥ 11.4 μmol/L for men for hyperhomocysteinemia [National Health and Nutrition Examination Survey III (Selhub et al. 1999)]; folate deficiency and hyperhomocysteinemia were very common, particularly in men (Table 1). We observed an inverse correlation between plasma cobalamin and tHcys concentrations (females: *r* = −0.26, *p* < 0.0001; males: *r* = −0.47, *p* < 0.0001).

Mean water As concentrations did not differ significantly by sex (Table 2). As expected, mean urinary creatinine concentrations were higher among males than among females, although this difference did not achieve statistical significance in this sample (Table 2). Mean concentrations of uAs were similar for males and females; however, uAs per gram creatinine (uAs/gCr) was significantly higher among females than among males. Women had significantly lower percentages of As in urine present as MMA (%MMA) and higher as DMA (%DMA), suggesting a higher As methylation capacity in women for the second methylation step (Table 2). Mean concentrations of As variables by cobalamin status are also shown in Table 2. Both water As and uAs/gCr were higher among cobalamin-deficient participants. Urinary %MMA was higher and %DMA was lower among cobalamin-sufficient participants than among cobalamin-deficient participants.

In bivariate analysis, plasma cobalamin was inversely correlated with both water As and uAs/gCr (Table 3). We noted a statistically significant positive correlation between cobalamin and %MMA (*r* = 0.18, *p* < 0.0001). Both %iAs and %DMA were weakly inversely correlated with cobalamin concentration, and these associations appeared to be the same in males and females (Table 3). In linear regression models (Table 4), cobalamin was significantly inversely associated with %iAs [unstandardized regression coefficient (b) = −0.10; 95% confidence interval (CI), −0.17 to −0.02; *p* = 0.01] and positively associated with %MMA (b = 0.12; 95% CI, 0.05 to 0.20; *p* = 0.001) after adjusting for age, sex, urinary creatinine, total uAs, and BMI. These associations were stronger among females than among males, but the difference was not statistically significantly (Table 4). Cobalamin was not significantly associated with urinary %DMA. Because the influence of cobalamin on As methylation could depend on an adequate supply of 5-methyl tetrahydrofolate (THF), we further examined associations between cobalamin and uAs metabolites by folate status (Table 4). Plasma cobalamin was significantly inversely associated with %iAs (b = −0.17; 95% CI, −0.30 to −0.03; *p* = 0.02) and positively associated with %MMA (b = 0.20; 95% CI, 0.11 to 0.30; *p* < 0.0001) only among folate-sufficient subjects (*p*-value for test of difference by folate status was 0.06 for %iAs and 0.04 for %MMA). We also examined associations between plasma cobalamin and uAs metabolites by water As concentrations ≤ 50 and > 50 μg/L (Table 4). The inverse association with %iAs was apparent only among those with water As concentrations ≤ 50 μg/L (b = −0.25; 95% CI, −0.40 to −0.09; *p* = 0.002); this difference was statistically significant (*p* = 0.005). Plasma cobalamin was significantly positively associated with %MMA in both strata of water As concentrations. Cobalamin was not significantly associated with urinary %DMA in either strata of water As.

### *In vitro* nonenzymatic As methylation by methylcobalamin

**Figure 2:**
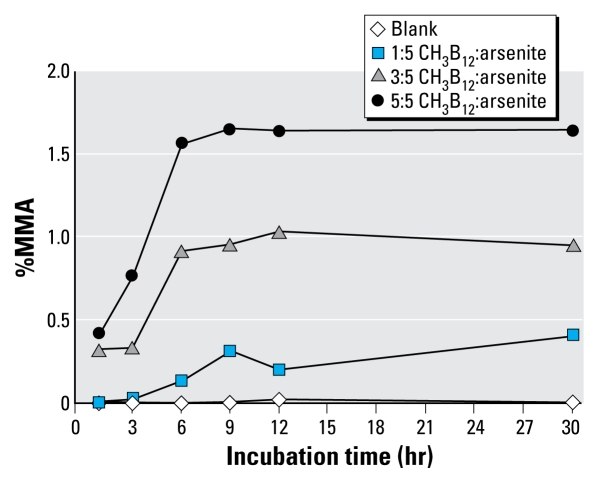
Methylation of arsenite by methyl-cobalamin by incubation time and molar ratio of CH_3_B_12_:AsIII at pH 7.4. As^III^ and CH_3_B_12_ were combined in Tris-HCl buffer in the presence of GSH, and aliquots were analyzed by HPLC-ICP-MS over time.

Incubation of arsenite with methylcobalamin in the presence of GSH resulted in the production of MMA (Figure 2). The amount of MMA increased with increasing molar ratios of CH_3_B_12_:arsenite and was highest with a molar ratio of 5:5 at a pH of 7.4. The amount of MMA also increased as the incubation period increased but appeared to plateau at approximately 9 hr. Very small amounts of DMA (i.e., < 0.05%) were also detected after 6–9 hr of incubation (data not shown).

## Discussion

This study complements our previous work in Bangladesh, in which we have investigated the contributions of folate and tHcys to variations in As methylation. In our study region in Araihazar, Bangladesh, the prevalence of cobalamin deficiency (8% of men and 13% of women < 151 pmol/L) is much lower than that of folate deficiency (57% of men and 39% of women < 9 nmol/L) (Gamble et al. 2005a). The current cross-sectional study, in which we oversampled cobalamin-deficient individuals, allowed us to examine a full range of plasma cobalamin values in relation to As metabolism. Our results showed that plasma cobalamin was inversely associated with %iAs and positively associated with %MMA in urine. Both of these associations were stronger among folate-sufficient individuals.

In addition to our previous cross-sectional study (Gamble et al. 2005b), six other studies (Chung et al. 2006; Hall et al. 2007; Heck et al. 2007; Li et al. 2008; Pilsner et al. 2009; Zablotska et al. 2008) have examined the role of cobalamin in relation to As metabolism or susceptibility to the health effects of As exposure. In our previous cross-sectional study of 1,016 Bangladeshi adults (Heck et al. 2007), we reported that dietary cobalamin intake as calculated from a food frequency questionnaire was inversely associated with %iAs and positively associated with %MMA in urine. These associations persisted after adjusting for age, sex, total caloric intake, cigarette smoking, and total uAs and are consistent with our current observations. In our recent cross-sectional study among 10,628 Bangladeshi adults chronically exposed to As, dietary cobalamin intake was not significantly associated with the odds of having skin lesions (Zablotska et al. 2008). Serum concentrations of cobalamin were also not associated with the odds of As-induced premalignant skin lesions in a case-control study in West Bengal, India (Chung et al. 2006). The interpretation of these findings is limited by the use of prevalent cases of skin lesions (Zablotska et al. 2008) and by the measurement of blood concentrations of cobalamin from samples obtained after the development of skin lesions (Chung et al. 2006). However, the findings are consistent with our nested case-control study in Bangladesh in which baseline folate deficiency and hyperhomocysteinemia, but not cobalamin, were associated with a roughly 70% increased risk for development of skin lesions at a 2-year follow-up visit (Pilsner et al. 2009). Our current findings, when compared with our previous cross-sectional study (Gamble et al. 2005b), suggest that associations between cobalamin concentrations and As metabolism may only be apparent when a full range of cobalamin concentrations (including deficient individuals) is examined. Our results also show that the higher urinary %MMA among cobalamin-sufficient adults than among cobalamin-deficient adults, although statistically significant, is modest. This may help to explain the lack of an association between cobalamin and As-induced skin lesions in previous studies.

In two other studies, researchers examined cobalamin in relation to As among pregnant Bangladeshi women. Li et al. (2008) reported that among 753 women, 60% of whom were cobalamin deficient, plasma cobalamin concentrations were not associated with uAs metabolites at gestational week 14. However, there was a suggestion that increasing plasma cobalamin concentrations were associated with decreased %iAs (mean %iAs for lowest and highest tertiles of plasma cobalamin were 13.7 and 11.7, respectively) and increased %DMA (mean %DMA for lowest and highest tertiles of plasma cobalamin were 76.5 and 79.2, respectively) among women with moderate uAs concentrations (58–209 μg/L). Li et al. (2008) suggested that the observed lack of a strong association between the nutrients examined and As metabolism may have been due to enhanced efficiency of As methylation during pregnancy related to increased synthesis of choline (Zeisel 2006). It is also possible that this result is related to prenatal folic acid supplement use. Hall et al. (2007), in a study of 101 maternal–newborn pairs, reported that maternal cobalamin concentration was significantly inversely associated with cord blood iAs^V^. In this study, maternal cobalamin was also inversely correlated with maternal urinary %iAs (*r* = −0.20, *p* = 0.05) and positively correlated with %DMA (*r* = 0.18, *p* = 0.09), although these associations were of borderline statistical significance (Hall et al. 2007).

Our findings of stronger associations between plasma cobalamin and uAs metabolites among women than among men are likely explained by the higher prevalence of cobalamin deficiency among these women. The modification of the associations between cobalamin and uAs metabolites by folate status is consistent with the role of 5-methyl THF as the methyl donor for the conversion of homocysteine to methionine by methionine synthase, the reaction for which cobalamin is a required cofactor and 5-methyl THF is a cosubstrate; methionine synthase activity is reduced when 5-methyl THF is limiting. It is unclear, however, why in the presence of adequate folate, cobalamin is not associated with increased %DMA. We hypothesize that cobalamin facilitates the first methylation step and that the reaction has a tendency to stop at MMA, although the underlying mechanism cannot be determined from our data. Interestingly, Drobna et al. (2006) found that shRNA silencing of AS3MT in HepG2 cells reduced the percentage of ^73^iAs^III^ methylated to DMA from 53% to 11%, whereas the synthesis of MMA actually increased from 10% to 13% of total ^73^As. Similarly, AS3MT knockout mice synethsize as much, if not more, MMA than wild-type mice (Drobna et al. 2009). These results strongly suggest the presence of an AS3MT-independent mechanism for MMA biosynthesis and prompted us to conduct an *in vitro* experiment.

Our *in vitro* methylcobalamin experiments, modeled after those of Zakharyan and Aposhian (1999), produced similar findings and support their suggestion that small amounts of MMA may be formed nonenzymatically. Our experiments differed from those of Zakharyan and Aposhian in that we examined the effect of pH and the molar ratio of CH_3_B_12_:arsenite on the production of MMA, and we omitted SAM, a possible source of methyl groups, from the reaction mixture. Not surprisingly, our results indicated that increasing the molar ratio of CH_3_B_12_:arsenite resulted in increased production of MMA. We also observed that the nonenzymatic methylation of arsenite by methylcobalamin was increased at pH levels closer to the normal pH of blood and other tissues than at pH 7.8. Whether the findings from our *in vitro* experiment, along with those of Zakharyan and Aposhian (1999), are relevant *in vivo* deserves further investigation.

The implications of our finding that cobalamin is associated with %MMA, but not with %DMA, are not entirely clear. However, a higher %MMA and lower %DMA in urine has been associated with increased risks of As-related skin lesions, skin cancer, bladder cancer, and peripheral vascular disease (Ahsan et al. 2007; Chen et al. 2003a, 2003b; Huang et al. 2008; Tseng 2007). These studies have measured %MMA^V+III^ in urine because of the difficulty of preserving the valence state. However, MMA^V^ is relatively nontoxic, whereas MMA^III^ is highly toxic (Styblo et al. 2002; Wang et al. 2007). Tseng (2007) suggested that the associations between MMA in urine and disease risks may actually be due to MMA^III^, which is not stable and is readily oxidized to MMA^V^. Our observed positive association between cobalamin and total %MMA in urine, taken together with recent evidence regarding the toxicity of MMA^III^, suggests that any influence of cobalamin on As-associated disease risk may not be favorable.

We measured plasma total cobalamin rather than methylmalonic acid or holotranscobalamin, two measurements that may be more sensitive indicators of cobalamin deficiency (Clarke et al. 2007; Obeid and Herrmann 2007). Although we may underestimate the prevalence of cobalamin deficiency, the statistical associations between cobalamin and uAs metabolites are dependent upon the ranking of individuals, not upon absolute concentrations of cobalamin. The interpretation of our findings is also limited by the expression of uAs metabolites as percentages, given that these values are relative to each other. However, meaningful patterns of association can still be detected when the data are expressed in this manner. We also did not have a measure of plasma choline or betaine, which may both be relevant sources of methyl groups for As methylation. In addition, we were not able to adjust for possible confounding by dietary intake of other nutrients that may influence As metabolism and also be correlated with cobalamin intake (such as methionine or creatine).

In summary, among this group of Bangladeshi adults with a high prevalence of cobalamin deficiency, cobalamin appeared to facilitate the methylation of iAs to MMA, particularly among women and among folate-sufficient individuals. The results of our *in vitro* experiment confirm that incubation of iAs with methylcobalamin can generate small amounts of MMA nonenzymatically. This finding is consistent with *in vivo* cell culture studies suggesting that alternative mechanisms for biosynthesis of MMA, independent of AS3MT, likely exist (Drobna et al. 2006, 2009). Given the recent evidence suggesting that MMA^III^ may be the most toxic As metabolite, our findings raise the possibility that, in contrast to folate, cobalamin supplementation may not favorably influence As metabolism.

## Figures and Tables

**Figure 1:**
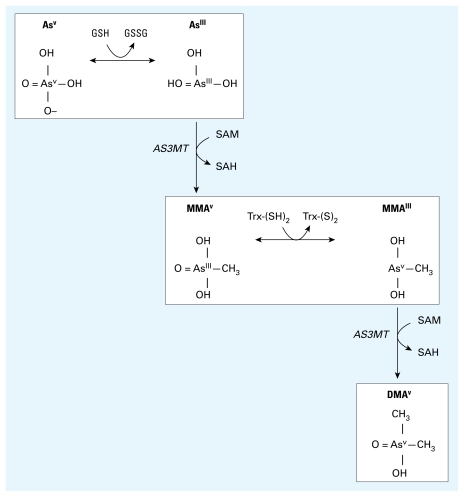
Arsenate is reduced to arsenite in a reaction thought to be dependent on GSH or other endogenous reductants. Arsenite then undergoes an oxidative methylation, with SAM as the methyl donor, forming MMA^V^ and SAH. MMA^V^ is reduced to MMA^III^ and then undergoes a subsequent oxidative methylation step to produce DMA^V^ and SAH.

**Table 1:** General characteristics of the study sample.

	Female (*n* = 479)		Male (*n* = 299)		
Characteristic	Mean ± SD	Median (range)	Mean ± SD	Median (range)	*p*-Value^a^
Age (years)	33.7 ± 8.8	32.0 (18.0–58.0)	42.9 ± 10.2	42.0 (21.0–67.0)	< 0.0001
Height (cm)	149.7 ± 5.2	149.6 (133.0–165.0)	161.8 ± 5.5	161.9 (145.4–176.7)	< 0.0001
Weight (kg)	45.3 ± 7.7	44.8 (27.8–73.7)	50.7 ± 8.3	50.0 (32.0–81.8)	< 0.0001
BMI (kg/m^2^)	20.2 ± 3.1	19.8 (14.3–33.3)	19.4 ± 2.8	18.8 (13.7–31.5)	0.0001
BMI < 18.5 (%)	31.6		45.6		< 0.0001
Current smoking (%)	2.9		59.1		< 0.0001
Current betel nut use (%)	30.6		40.9		0.003
Education (years)	3.1 ± 3.5	2.0 (0–14)	3.8 ± 3.9	3.0 (0–16.0)	0.02
Type of housing (%)					
Thatched	7.7		7.4		0.49
Corrugated tin	77.7		80.9		
Other	14.6		11.7		
Water As > 50 μg/L (%)	60.5		57.9		0.46
Water As > 10 μg/L (%)	81.4		83.3		0.51
Plasma cobalamin (pmol/L)	217.3 ± 114.6	175.9 (73.8–1171.8)	241.7 ± 117.2	197.7 (73.8–678.7)	0.0006
Plasma cobalamin < 151 pmol/L (%)^b^	26.5		19.4		0.02
Plasma cobalamin < 185 pmol/L (%)^c^	57.6		46.8		0.003
Plasma folate (nmol/L)	13.2 ± 9.3	10.1 (2.9–66.2)	9.4 ± 7.1	7.2 (2.6–52.7)	< 0.0001
Plasma folate < 9.0 nmol/L (%)^b^	42.0		69.2		< 0.0001
Plasma tHcys (μmol/L)	9.7 ± 5.0	8.8 (0.2–46.8)	16.5 ± 11.8	13.4 (3.0–90.1)	< 0.0001
Hyperhomocysteinemia (%)^d^	29.7		65.1		< 0.0001

a For test of difference by sex, based on Wilcoxon’s rank-sum test for continuous variables and chi-square or Fisher’s exact test for categorical variables.

b Based on reference values from Christenson et al. (1985).

c Based on reference values from Pfeiffer et al. (2005).

d Defined as ≥ 10.4 μmol/L for women and as ≥ 11.4 μmol/L for men.

**Table 2:** Mean concentrations of As variables and urinary creatinine, by sex and cobalamin status.

Variable	Female (*n* = 479) [mean (95% CI)]	Male (*n* = 299) [mean (95% CI)]	*p*-Value^a^	Cobalamin sufficient (*n* = 362) [mean (95% CI)]	Cobalamin deficient (*n* = 416) [mean (95% CI)]	*p*-Value^b^
Water As (μg/L)	104.8 (95.3–114.3)	101.3 (89.6–113.0)	0.59	95.7 (85.0–106.4)	110.2 (100.0–120.3)	0.006
Urinary creatinine (mg/dL)	58.9 (55.2–62.7)	66.4 (60.8–72.1)	0.12	62.9 (58.1–67.6)	60.9 (56.6–65.1)	0.49
uAs (μg/L)	141.1 (130.3–151.9)	141.7 (125.0–158.5)	0.18	136.8 (123.5–150.1)	145.3 (132.4–158.1)	0.16
uAs/gCr	293.3 (271.0–315.6)	242.9 (221.2–264.6)	0.002	263.5 (237.8–289.2)	283.0 (262.7–303.2)	0.004
%iAs	15.7 (15.0–16.4)	15.0 (14.4–15.6)	0.54	15.7 (14.9–16.5)	15.2 (14.6–15.8)	0.92
%MMA	10.7 (10.3–11.1)	14.5 (13.9–15.1)	< 0.0001	13.2 (12.7–13.7)	11.2 (10.8–11.7)	< 0.0001
%DMA	73.6 (72.8–74.4)	70.5 (69.5–71.4)	< 0.0001	71.1 (70.1–72.0)	73.6 (72.8–74.3)	< 0.0001
MMA:iAs Ratio	0.79 (0.75–0.83)	1.07 (1.0–1.1)	< 0.0001	0.99 (0.93–1.04)	0.82 (0.78–0.86)	< 0.0001
DMA:MMA Ratio	8.5 (8.1–9.0)	5.7 (5.3–6.0)	< 0.0001	6.5 (6.1–6.9)	8.2 (7.8–8.7)	< 0.0001

a Wilcoxon’s rank sum test for sex difference.

b Wilcoxon’s rank sum test for difference by cobalamin status.

**Table 3:** Spearman’s correlation coefficients between cobalamin and As variables by sex.

		Sex	
Variable	Total sample	M (*n* = 299)	F (*n* = 479)
Water As (μg/L)	−0.08*	−0.04	−0.11*
Urinary creatinine (mg/dL)	0.07	0.09	0.04
uAs (μg/L)	−0.03	0.04	−0.08
uAs/gCr	−0.12#	−0.04	−0.15**
Urinary %iAs	−0.06	−0.02	−0.09
Urinary %MMA	0.18##	0.10	0.18#
Urinary %DMA	−0.08*	−0.05	−0.05

* *p* < 0.05.

** *p* < 0.01.

# *p* < 0.001.

## *p* < 0.0001.

**Table 4:** Parameter estimates for effect of increasing log plasma cobalamin on percentage of uAs metabolites.

	%iAs			%MMA			%DMA		
	Parameter estimate (95% CI)	*p-*Value^a^	*p-*Value^b^	Parameter estimate (95% CI)	*p-*Value^a^	*p-*Value^b^	Parameter estimate (95% CI)	*p-*Value^a^	*p-*Value^b^
Total (*n* = 766)^c^	−0.10 (−0.17 to −0.02)	0.01		0.12 (0.05 to 0.20)	0.001		−0.01 (−0.03 to 0.006)	0.16	

Females (*n* = 471)^d^	−0.12 (−0.22 to −0.01)	0.03	0.45	0.17 (0.07 to 0.26)	0.0009	0.14	−0.02 (−0.04 to 0.009)	0.19	0.69
Males (*n* = 295)^d^	−0.06 (−0.17 to 0.05)	0.31		0.05 (−0.07 to 0.17)	0.38		−0.01 (−0.04 to 0.02)	0.51	

Folate sufficient (*n* = 365)^c^	−0.17 (−0.30 to −0.03)	0.02	0.06	0.20 (0.11 to 0.30)	< 0.0001	0.04	−0.02 (−0.05 to 0.01)	0.22	0.54
Folate deficient (*n* = 401)^c^	−0.02 (−0.09 to 0.05)	0.64		0.05 (−0.07 to 0.16)	0.41		−0.008 (−0.03 to 0.02)	0.54	

Water As < 50 μg/L (*n* = 310)^c^	−0.25 (−0.40 to −0.09)	0.002	0.005	0.16 (0.02–0.30)	0.03	0.47	0.003 (−0.02 to 0.03)	0.86	0.24
Water As > 50 μg/L (*n* = 456)^c^	−0.0008 (−0.08 to 0.07)	0.98		0.10 (0.02–0.18)	0.02		−0.02 (−0.05 to 0.008)	0.16	

a *p*-Value for the test of significance of the parameter estimate.

b *p*-Value from the Wald test for differences in stratum-specific estimates (male vs. female, folate sufficient vs. deficient, and water As < 50 μg/L vs. > 50 μg/L).

c Adjusted for log age (continuous), sex, log urinary creatinine (continuous), log total uAs (continuous), and log BMI (continuous).

d Adjusted for all variables in (*^c^*) except sex.
